# An Electrochromic Nickel Phosphate Film for Large-Area Smart Window with Ultra-Large Optical Modulation

**DOI:** 10.1007/s40820-022-01002-4

**Published:** 2023-01-11

**Authors:** Pengyang Lei, Jinhui Wang, Yi Gao, Chengyu Hu, Siyu Zhang, Xingrui Tong, Zhuanpei Wang, Yuanhao Gao, Guofa Cai

**Affiliations:** 1 Key Laboratory for Special Functional Materials of Ministry of Education, National and Local Joint Engineering Research Center for High-Efficiency Display and Lighting Technology, School of Materials and Engineering, and Collaborative Innovation Center of Nano Functional Materials and Applications, Henan University, Kaifeng, 475004 People’s Republic of China; 2 Key Laboratory of Micro-Nano Materials for Energy Storage and Conversion of Henan Province, Institute of Surface Micro and Nano Materials, College of Chemical and Materials Engineering, Xuchang University, Xuchang, 461000 Henan People’s Republic of China

**Keywords:** Electrochromism, Transition metal phosphates, Optical modulation, Smart window, Energy storage

## Abstract

Exploring materials with high electrochemical activity is of keen interest for electrochemistry-controlled optical and energy storage devices. However, it remains a great challenge for transition metal oxides to meet this feature due to their low electron conductivity and insufficient reaction sites. Here, we propose a type of transition metal phosphate (NiHPO_4_·3H_2_O, NHP) by a facile and scalable electrodeposition method, which can achieve the capability of efficient ion accommodation and injection/extraction for electrochromic energy storage applications. Specifically, the NHP film with an ultra-high transmittance (approach to 100%) achieves a large optical modulation (90.8% at 500 nm), high coloration efficiency (75.4 cm^2^ C^−1^ at 500 nm), and a high specific capacity of 47.8 mAh g^−1^ at 0.4 A g^−1^. Furthermore, the transformation mechanism of NHP upon electrochemical reaction is systematically elucidated using in situ and ex situ techniques. Ultimately, a large-area electrochromic smart window with 100 cm^2^ is constructed based on the NHP electrode, displaying superior electrochromic energy storage performance in regulating natural light and storing electrical charges. Our findings may open up new strategies for developing advanced electrochromic energy storage materials and smart windows.

**Supplementary Information:**

The online version contains supplementary material available at 10.1007/s40820-022-01002-4.

## Highlights

- Transition metal phosphates were first developed for the electrochromic application.
- The obtained NiHPO_4_·3H_2_O film achieves an ultra-large optical modulation of 90.8%, and the electrochromic mechanism is systematically elucidated using in situ and ex situ techniques.
- A large-area electrochromic smart window with 100 cm^2^ is constructed, which displays superior performances in regulating natural lighting and storing electrical charges.

**Supplementary Information:**

The online version contains supplementary material available at 10.1007/s40820-022-01002-4.

## Graphical abstract

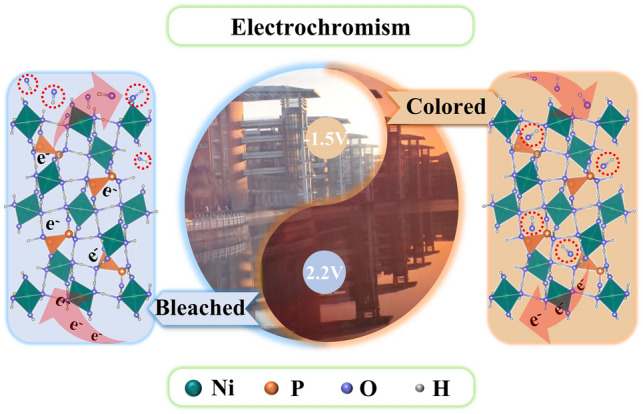

## Introduction

Electrochromic (EC) technology is a vital component of energy-efficient utilization and socially sustainable development. The EC smart window can save about 20–40% energy consumption in lighting and temperature control of buildings [1–3]. In addition, the dynamic regulation characteristics of the EC smart window can provide the occupants with controlled natural lighting while protecting their privacy [4–6]. Transition metal oxides such as nickel oxide [7], tungsten oxide [8], and titanium oxide [9] are considered promising EC materials as the core configuration of EC smart windows. Among them, nickel (Ni)-based EC materials have received broad research due to their low cost and neutral coloring feature [10–12]. Nevertheless, the optical modulation of most Ni-based materials is still limited due to their low electron conductivity and insufficient reaction sites.

To overcome this issue, many efforts have been devoted to designing Ni-based EC materials to improve their electrochemical activity. For instance, Zeng et al. reported a nitrogen–carbon co-doped NiO electrode with enhanced electrical conductivity through the one-step pyrolysis of Ni-MOF film, showing an optical modulation of 68% at 580 nm and a 71.6% of transmittance in the bleached state [13]. Cai et al*.* synthesized the 1D π-d conjugated coordination polymer (Ni-BTA) film with high conductivity by introducing conjugated structures, which exhibit 61.3% of optical contrast at 500 nm and about 90% of transmittance in the bleached film [14]. Besides, the preheating treatment method was presented to avoid the transformation from α-Ni(OH)_2_ film with high electrochemical activity to β-Ni(OH)_2_, which exhibits a large optical modulation of 78.6% at 550 nm with 89% of transmittance in the bleached state [15]. Although these strategies aim to enhance the EC performances of Ni-based materials, the optical modulation is still unsatisfactory as their low transmittance in the bleached state and the restricted electrochemical activity. In addition, the high cost and complex preparation technology are limiting the practical application of uniform EC films over a large area, such as solvothermal and hydrothermal methods [16–18]. Therefore, exploring appropriate Ni-based EC materials and synthesis technologies are highly desirable to develop high-performance large-area EC smart windows.

From the perspective of rational component design, exploring electrochromic materials with high electrochemical activity is a judicious and powerful approach for dramatically improving their performance. In this regard, transition metal phosphates (TMPs) with layers of connected metal–oxygen hexahedron and phospho-oxygen tetrahedron are promising candidates [19–21]. Originated from the properties of Lewis acid, the coordinated HPO_4_^2−^ anions in TMPs can be used as trapping points to enhance the adsorption capacity of OH^−^, resulting in the improved electron conductivity and electrochemical activity of TMPs in alkaline electrolytes, compared to that of transition metal oxides [22, 23]. Recent studies on TMPs have confirmed the broad applications in various fields such as electrochemical energy storage and electrocatalysis [24, 25]. Nevertheless, there has been little discussion about TMPs-based materials in the EC field so far, especially the EC mechanism of TMPs which is not clear yet.

In this work, we proposed and demonstrated a large-area nickel phosphate (NiHPO_4_·3H_2_O, NHP) EC film with homogeneous nanoparticles by a facile and scalable electrodeposition method. As a bifunctional film with electrochromism and energy storage, the NHP film showed large optical modulation with nearly 100% transmittance in the bleached state, high coloration efficiency, and comparable gravimetric capacity. The reaction mechanism of NHP film was revealed by in situ and ex situ techniques, which indicate that the Ni^2+^ is reversibly converted to Ni^3+^ during the electrochemical process. Besides, we further integrated a prototype of the EC smart window composed of the NHP-based EC layer and TiO_2_-based ion storage layer. The EC smart window with 10 × 10 cm^2^ not only controlled the transmittance intensity of natural light into the building, but also recovered electric energy to accomplish the energy reutilization. Overall, our work provides an opportunity for the TMPs to implement large-area high-performance EC smart windows.

## Experimental Section

### Materials

Nickel nitrate hexahydrate (Ni(NO_3_)_2_·6H_2_O, ≥ 98%) was purchased from Sinopharm Chemical Reagent. Sodium hypophosphite monohydrate (NaH_2_PO_2_·H_2_O, ≥ 99%) and titanium oxide (TiO_2_, ≥ 99.8%) were purchased from Aladdin Industrial Corporation. Poly(vinyl alcohol) (PVA, Mw = 89,000–98,000, ≥ 99%) was purchased from Sigma-Aldrich. Potassium hydroxide (KOH, ≥ 85%) was purchased from Kermel Chemical Reagent. All chemicals were not further purified, and the deionized water was prepared in a water purifier (Milli-Q 18 MΩ, Millipore Corp).

### Fabrication of NiHPO_4_·3H_2_O Nanoparticle Films

The electrodeposition electrolyte solution was obtained by dissolving Ni(NO_3_)_2_·6H_2_O (0.2385 g) and NaH_2_PO_2_·H_2_O (0.1053 g) in 100 mL of the mixture of deionized water and ethanol (*v*/*v* = 1:1). The glass coated with fluorine-doped tin oxide (FTO) and polyethylene terephthalate (PET) coated with indium tin oxide (ITO) were used as transparent conductive electrodes and successively ultrasonic cleaned with acetone, deionized water and ethanol for 15 min. In the three-electrode system, the FTO (2 × 5 cm^2^), platinum (Pt) foil and Ag/AgCl were used as the working electrode, counter electrode, and reference electrode, respectively. The electrodeposition process was performed by cyclic voltammetry within a potential window of − 1.2 to 0.2 V at a scan rate of 20 mV s^−1^ for 4 cycles. The samples were rinsed with deionized water and then dried at 60 ℃ for 12 h in an oven. Finally, the NiHPO_4_·3H_2_O nanoparticle films were successfully prepared.

### Fabrication of TiO_2_ Nanoparticle Films

The ion storage layer of TiO_2_ film on the FTO substrate was prepared by electrostatic spray deposition (ESD) technology. In order to obtain the homogeneous ESD solution, the purchased TiO_2_ nanoparticles were diluted to 1 mg mL^−1^ with ethanol and deionized water, and then, the suspension was treated with ultrasonic for 1 h. Finally, the as-prepared ESD precursor solution was transferred to a syringe with a metal nozzle. The TiO_2_ nanoparticle film on FTO conductive glass was obtained by spraying 0.5 mL solution at 18 kV high voltage using the ESD technology.

### Fabrication of the 1 M KOH/PVA Gel

Firstly, a solution was prepared by dissolving 1.5 g polyvinyl alcohol (PVA) in 20 mL deionized water at 85 ℃ in an oil bath. Then, 1.4 g potassium hydroxide (KOH) was dissolved in the cooled PVA solution under stirring. Ultimately, the KOH/PVA gel-type electrolyte was successfully obtained.

### Assembly of Semi-Solid-State Device

The semi-solid-state device was assembled using NiHPO_4_·3H_2_O nanoparticle film as an electrochromic layer, TiO_2_ nanoparticle film as an ion storage layer, and 1 M KOH/PVA gel as an electrolyte. The mounting tape (VHB 4010, 3 M) with a thickness of 1.5 mm was applied to connect NiHPO_4_·3H_2_O layer and TiO_2_ layer to create a space for holding the gel electrolyte. Finally, the semi-solid-state electrochromic device was sealed with an epoxy resin adhesive.

### Materials Characterization

The crystal structure and material composition were characterized by X-ray diffraction (XRD, D8-ADVANCE). The microstructure and elemental mapping of the samples were characterized using a field emission scanning electron microscope (FESEM, Nova NanoSEM 450) and a transmission electron microscope (TEM, JEM-2100). The in situ Raman spectra of the material at different states were obtained on Laser microscopic Raman spectroscopy (Raman, Renishaw inVia) with a laser wavelength of 532 nm. The FTIR spectrum was conducted by a Frontier IR/FIR STA 8000 spectrometer (PerkinElmer, USA) in the range of 400–2000 cm^−1^ with an attenuated total reflection detector. The X-ray photoelectron spectroscopy (XPS) was performed by an AXIS ULTRA X-ray photoelectron spectrometer to determine the element’s composition and valence of the sample.

### Electrochemical and Electrochromic Characterization

Electrochemical and electrochromic measurements were implemented in the three-electrode system. The NiHPO_4_·3H_2_O film was used as the working electrode, Ag wire served as the reference electrode, Pt wire worked as the counter electrode, and 1 M KOH aqueous solution as the electrolyte. The in situ spectrum response accompanied by cyclic voltammetry (CV), chronoamperometry (CA), and galvanostatic charge–discharge tests was carried out at 500 nm via the integration of the Autolab electrochemical workstation (PGSTAT302N) and a Shimadzu UV-3600 Plus spectrophotometer. CV curves were measured at 10 mV s^−1^ with the potential range from 0 to 0.7 V (*vs.* Ag^+^/Ag), and galvanostatic discharge–charge measurements with different current densities were performed in the potential window of 0 to 0.5 V (*vs.* Ag^+^/Ag). The transmittance spectra of the colored and bleached states of NiHPO_4_·3H_2_O and TiO_2_ films were evaluated by the aforesaid spectrophotometer in the wavelength range of 300–800 nm. Furthermore, the transmittance of FTO and air was selected as the baselines when conducting the spectral tests on film and device, respectively.

## Results and Discussion

### Preparation and Characterization of NHP Film

**Fig. 1:**
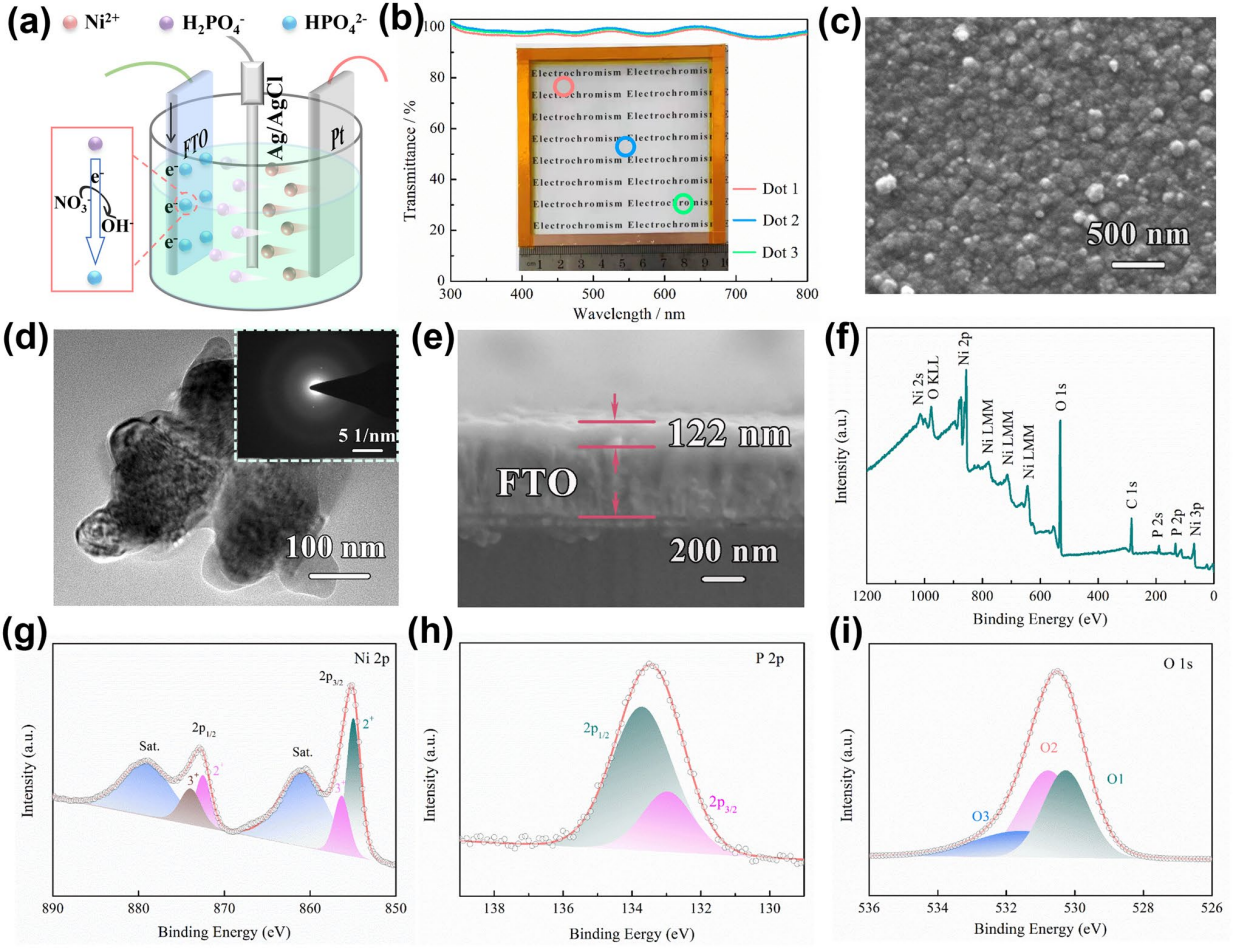
**a** Schematic illustration of the NHP film obtained by cyclic voltammetric electrodeposition from − 1.2 to 0.2 V (*vs.* Ag^+^/Ag) at 20 mV s^−1^ in a three-electrode system. **b** Transmission spectra of three different points selected from a 10 × 10 cm^2^ NHP film as shown in the inset. **c** SEM images of NHP film prepared by electrodeposition on FTO substrates, **d** TEM image and the corresponding SAED pattern (inset) of the NHP nanomaterial, and **e** represents the cross-sectional SEM of NHP film. **f** XPS spectrum of the NHP film. **g** Ni 2*p*, **h** P 2*p* and **i** O 1*s* spectrum of the NHP film

The NiHPO_4_·3H_2_O (NHP) film was prepared by a facile and scalable electrodeposition method. Detailly, the cyclic voltammetry (CV) technique was utilized to control the deposition process (Fig. S1). The NO_3_^−^ is reduced on the surface of FTO to produce OH^−^, and OH^−^ further reacts with H_2_PO_2_^2−^ to generate HPO_4_^2−^ (Fig. 1a). In the meantime, the positively charged Ni^2+^ in the electrolyte migrates to the negatively charged FTO surface, forming an NHP film directly on the FTO surface with HPO_4_^2−^ [19, 26]. Therefore, the deposition mechanism can be summarized in the following formula:

$$ {\text{NO}}_{3}^{ - } + 2{\text{e}}^{ - } + {\text{H}}_{2} {\text{O}} \to {\text{NO}}_{2}^{ - } + 2{\text{OH}}^{ - } $$

$$ {\text{H}}_{2} {\text{PO}}_{2}^{ - } + {\text{OH}}^{ - } \to {\text{HPO}}_{4}^{2 - } + {\text{H}}_{2} {\text{O}}^{ } $$

$$ {\text{Ni}}^{2 + } + {\text{HPO}}_{4}^{2 - } + 3{\text{H}}_{2} {\text{O}} \to {\text{NiHPO}}_{4} \cdot 3{\text{H}}_{2} {\text{O}} $$

A transparent NHP film on FTO glass with a large area of 10 × 10 cm^2^ was obtained by setting four CV deposition cycles. The transmittance at three different sites was investigated and identically reached up to ~ 99.5% at 500 nm, manifesting the spatially homogeneous fabrication of the electrodeposition method (Fig. 1b). Scanning electron microscopy (SEM) image further demonstrates that the highly identical transmittance originates from the formation of uniform nanoparticles in NHP film (Fig. 1c). The diameter of NHP nanoparticles is around 100–120 nm confirmed by TEM (Fig. 1d), which is in good agreement with SEM results. The thickness of NHP film was controlled by only ~ 122 nm (Fig. 1e), far smaller than that of reported Ni-based EC materials (> 500 nm) [10, 16, 27]. This can be attributed to the high ionic conductivity (σ) of the electrodeposition electrolyte due to the introduction of phosphates (*σ*_with *p*_ = 0.99 mS cm^−1^), compared with that without phosphates (*σ*_without *p*_ = 0.62 mS cm^−1^) as shown in Fig. S2. The selected area electron diffraction (SAED) pattern presents the halo rings without any identifiable diffraction spots, demonstrating the amorphous nature of the NHP film (inset of Fig. 1d). Furthermore, the diffraction peaks except for the FTO were hardly observed in XRD measurement (Fig. S3), further confirming this viewpoint. The amorphous structure generally endows the material with enhanced charge transfer properties and improved electrochemical reaction kinetics [28]. The presence and elemental distribution of Ni, P, and O in NHP film were additionally identified by energy-dispersive X-ray spectroscopy (EDS, Fig. S4). To elucidate the chemical composition and oxidation state of NHP film, XPS analysis was performed. The survey spectrum of as-prepared NHP film illustrates the existence of Ni, P, O, and C elements (Fig. 1f). The Ni 2*p* spectrum in Fig. 1g is well fitted into two spin–orbit doublets of Ni^2+^ (at 854.8 and 872.5 eV) and Ni^3+^ (at 856.2 and 873.9 eV) accompanied by two satellite peaks (at 860.8 and 879.1 eV) [29]. According to the P 2*p* spectrum (Fig. 1h), two peaks at 132.9 and 133.7 eV correspond to the 2*p*_3/2_ and 2*p*_1/2_ in phosphorous species [30]. Besides, oxygen-contained bonds are measured and the O 1*s* spectrum is divided into O1 (530.2 eV), O2 (530.7 eV), and O3 (531.5 eV) species, which are assigned to Ni–O–P, P–O–H, and H_2_O, respectively (Fig. 1i) [22]. Overall, these results indicate that large-area NHP nanoparticles film is successfully fabricated by the one-step electrodeposition method.

### Electrochromic Performance of NHP Film

**Fig. 2:**
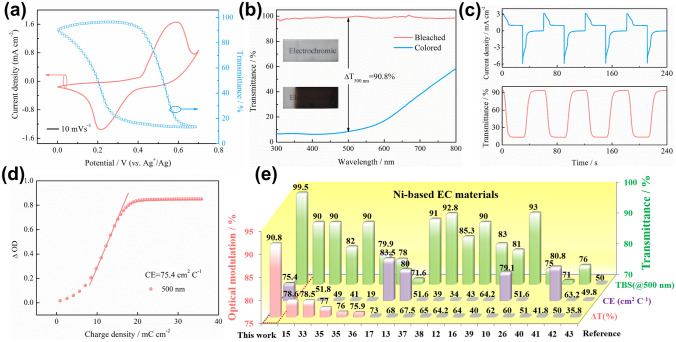
Electrochemical and electrochromic performance of the NHP film. **a** CV curve and in situ response spectrum at 500 nm measured at 10 mV s^−1^ in 1 M KOH electrolyte. **b** Optical transmittance spectra of the NHP film in 300–800 nm at colored (0.7 V *vs.* Ag^+^/Ag) and bleached (0 V *vs.* Ag^+^/Ag) states. Insets display digital photographs of the colored and bleached NHP film. **c** The profiles of current density and corresponding in situ response spectrum in 500 nm recorded by applying alternating potentials of 0 and 0.7 V (*vs.* Ag^+^/Ag) each for 30 s. **d** Optical density as a function of the charge density at 500 nm for the NHP film. **e** Histogram of optical modulation, coloration efficiency (CE) and transmittance of bleached state (TBS) of NHP film compared with other nickel-based EC materials

Electrochemical and electrochromic performances of the as-prepared NHP film were evaluated in a 1 M KOH electrolyte by the spectroelectrochemical three-electrode system. The CV measurement was performed on the NHP film in a potential window of 0 to 0.7 V (*vs.* Ag^+^/Ag) at 10 mV s^−1^ (Fig. 2a). One pair of apparent redox peaks at around 0.58 and 0.21 V (*vs.* Ag^+^/Ag) is observed, which can be assigned to the transition between Ni^2+^ and Ni^3+^, indicating a standard battery-like behavior [31–33]. Meanwhile, the in situ spectral response at 500 nm was recorded along with the CV measurement. The transmittance of the NHP film gradually decreases in the OH^−^ injecting process from 0.3 to 0.7 V (*vs.* Ag^+^/Ag) and then dynamically restores to the initial state with the OH^−^ extracting when the potential returns to 0 V (*vs.* Ag^+^/Ag), showing superior electrochemical and optical reversibility. As a crucial parameter for electrochromic materials, optical modulation (Δ*T*) is the difference between the transmittance of colored state (*T*_c_) and bleached state (*T*_b_) at a single wavelength [34]. It can be seen that the bleached NHP film always maintains close to 100% transmittance in the wavelength range of 300–800 nm (Fig. 2b), while the colored film possesses a transmittance of only 8.7% at 500 nm. Hence, an impressive optical modulation of up to 90.8% can be achieved, benefiting from the ultra-thin thickness and high electrochemical activity of the NHP film, whereas only 69.5% can be reached for the phosphorus-free film (Fig. S5a). In particular, the NHP film also shows more than 90% of optical modulation at the ultraviolet (UV) band from 300 to 400 nm, enabling controllable utilization of UV light. As shown in the inset of Fig. 2b, the ultra-large optical modulation is also reflected by the digital photographs in which the bleached NHP film delivers high visible transparency and a neutral characteristic in the colored state. Compared with the CV curve of Ni-based film without phosphorus source, it can be observed that the excellent modulation ability can be achieved by NHP film, resulting in the increase of the electrochemically active sites and the sufficient electrochromic reaction (Fig. S5b). To verify the feasibility of fabrication on different substrates, NHP film was electrodeposited on a flexible ITO/PET substrate with an optical modulation of 76.7% at 500 nm (Fig. S6a). The digital photographs of curved NHP film present stable electrochromism in both colored and bleached states (Fig. S6b).

The switching characteristic of the NHP film was further investigated by alternately applying the potentials of 0.7 and 0 V (*vs.* Ag^+^/Ag) with a duration of 30 s. The electrochromic kinetics and reversibility are reflected by the repeating current and transmittance responses at 500 nm (Fig. 2c). Coloring time (*t*_c_) and bleaching time (*t*_b_) of the NHP film are determined according to the time taken for 90% of the maximum optical modulation during the switching process [35]. The calculated result from the in situ spectrum shows that *t*_c_ and *t*_b_ are 7.1 and 9.6 s, respectively, which are shorter than the reported NiO film with 63.6% optical modulation (11.5 and 9.5 s) [17]. The rapid response behavior of the NHP film may ascribe to the reduced charge transfer resistance (*R*_ct_) owing to the introduced phosphorus source and the short electron transport path in the film with low thickness (Fig. S7, *R*_ct with *p*_ = 33.5 Ω, *R*_ct without *p*_ = 38.6 Ω). Besides, the effects of the electrodeposition cycles on the electrochromic properties of NHP films were further investigated (Table S1). It is worth noting that the NHP film prepared by four electrodeposition cycles exhibits an optical modulation of 90.8%. However, the performances of optical modulation and switching time were decayed when further increasing electrodeposition cycles. It is mainly attributed to the increase in the thickness of NHP film, resulting in the reduction of transmittance at the bleached state and the extended ion and electron transport path. Coloration efficiency (CE) is closely related to Δ*T* and current density, which is defined as the change in optical density (ΔOD) with respect to the unit charge density (Δ*Q*) during the color-switching process. The calculation formula is as follows:

$$ {\text{CE}}\left( \lambda \right) = \frac{\Delta OD}{{\Delta Q}} = \log {{\left( {\frac{{T_{{\text{b}}} }}{{T_{{\text{c}}} }}} \right)} \mathord{\left/ {\vphantom {{\left( {\frac{{T_{{\text{b}}} }}{{T_{{\text{c}}} }}} \right)} {\Delta Q}}} \right. \kern-0pt} {\Delta Q}} $$

where *T*_c_ and *T*_b_ refer to the transmittance of colored and bleached states, respectively. In general, a high CE means that a large optical modulation could be realized by only a small amount of charge. The CE of the NHP film calculated from the slope of the linear fit region in OD versus *Q*/*A* plots is 75.4 cm^2^ C^−1^ at 500 nm (Fig. 2d), which is relatively higher than that of previously reported Ni-based EC materials (most of them are less than 60 cm^2^ C^−1^, Fig. 2e) [10–13, 15–17, 27, 36–45]. Moreover, the optical modulation and transmittance of the bleached state in this NHP film are superior to the previous literature on Ni-based EC materials (Δ*T* is generally less than 80%, Fig. 2e). The optical memory represents sustainable management of solar light, which is crucial to the energy-saving of smart windows. To this point, it is worth noting that the NHP film can maintain optical stability in colored and bleached states over 5000 s with a transmittance decay of ~ 12.6% and 1.2% (Fig. S8). Additionally, the NHP film achieves robust cycling stability and retains 76.1% of optical modulation over 300 electrochemical cycles, while the coloration and bleaching response times are changed to 4.4 and 12.6 s (Fig. S9). The reduced optical modulation is mainly caused by the structural deformation in process of ions intercalation/deintercalation.

**Fig. 3:**
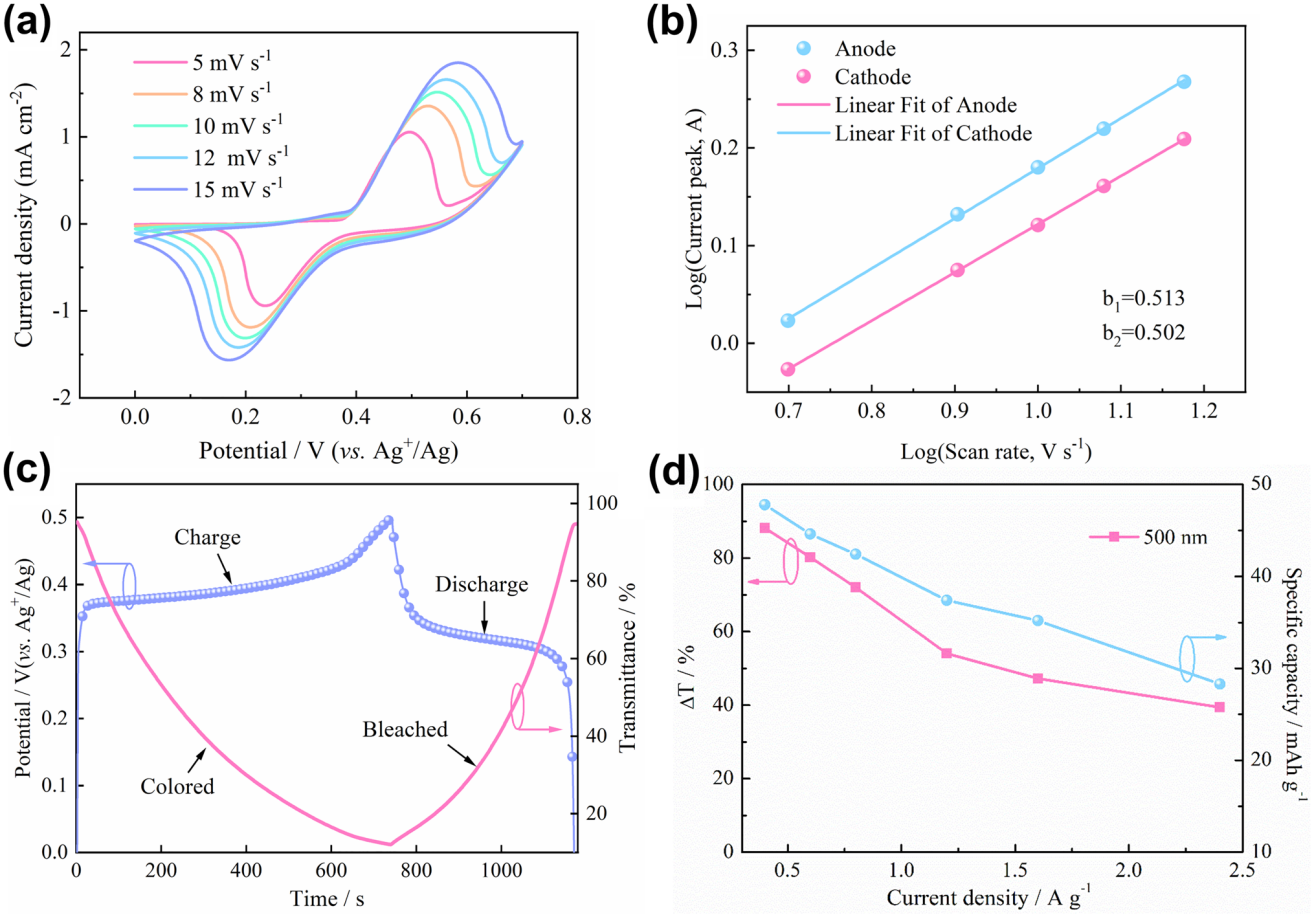
**a** CV curves of NHP film at different scan rates from 5 to 15 mV s^−1^ between 0 and 0.7 V (*vs.* Ag^+^/Ag). **b** Calculation of b value according to the relationship between log(*v*) and log(*A*) in the CV measurements of NHP film. **c** Galvanostatic charge–discharge profile at 0.4 A g^−1^ and corresponding in situ response spectrum at 500 nm of the NHP film. **d** Optical modulation and specific capacity of the NHP film as a function of current density

In order to assess the electrochemical reaction kinetics of the NHP electrode, the charge storage mechanism was investigated using CV measurements at a different scan rate from 5 to 15 mV s^−1^ (Fig. 3a). The current density of redox peaks increases synchronously with the increasing scan rate. The redox peaks are still maintained well at a higher scan rate of 15 mV s^−1^, manifesting rapid ion transport and low internal resistance in the electrode material [46]. Meanwhile, the superior reversibility of the NHP film can be concluded by the symmetry of redox peaks. Furthermore, the electrochemical contributions of the surface-capacitive and diffusion-controlled process can be qualitatively calculated according to the following formula:

$$ i = av^{b} $$

Here, *i* and *v* are the peak current and scan rate, respectively, and *a* and *b* represent the constants. The electrochemical process is controlled by the surface faradic reaction when *i* has a linear relationship with the *v* (*b* = 1), indicating the pseudocapacitance behavior of the material. When *i* is proportional to the square root of the *v* (*b* = 0.5), the electrochemical process is dominated by the standard diffusion behavior [29]. The *b* value of the NHP electrode calculated by fitting log(*i*) and log(*v*) curves are 0.513 (*b*_1_) and 0.502 (*b*_2_), respectively, corresponding to anodic and cathodic current peaks (Fig. 3b). It indicates that the electrochemical reaction of the NHP electrode is mainly dominated by the diffusion process. The high consistency of the b_1_ and b_2_ further corroborates the excellent reversibility of the electrochemical process [46].

Considering the good battery-like energy storage behaviors of the NHP film, we further quantitatively evaluated its energy storage performance by galvanostatic charge–discharge (GCD) measurement from 0 to 0.5 V (*vs.* Ag^+^/Ag) (Fig. S10). The GCD curves with different current densities have a pair of obvious platforms at 0.36–0.42 and 0.34–0.29 V (*vs.* Ag^+^/Ag), which correspond to the charging (oxidation) and discharging (reduction) processes of the NHP film. The specific capacities of the NHP film were 47.8, 44.6, 42.4, 37.4, and 35.2 mAh g^−1^ at current densities of 0.4, 0.6, 0.8, 1.2, and 1.6 A g^−1^, respectively, which are higher than the Ni-based materials used as energy storage (33.6 mAh g^−1^ at 0.25 A g^−1^) [12]. Even when the current density is expanded to 2.4 A g^−1^, 60% of the specific capacity (28.3 mAh g^−1^) can still be remained in comparison to the capacity at 0.4 A g^−1^, showing satisfactory rate capability of the NHP film. Furthermore, the NHP film presents changed Coulomb efficiency from 67.1 to 97.9% at different current densities, manifesting its good electrochemical reversibility (Fig. S11). The energy density of NHP film was calculated to be 15.3 Wh kg^−1^ at a power density of 127.5 W kg^−1^ and a maximum power density of 777.8 W kg^−1^ at an energy density of 9.1 Wh kg^−1^ as shown in Ragone plots in Fig. S12. Besides, the durability of the NHP film was provided by comparing the capacity change before and after cycling (Fig. S13). It demonstrates that 79.8% of capacity can be maintained, which is in agreement with the change in optical modulation (76.1%) of the NHP film. These results indicate that the NHP film can efficiently store energy, which is extremely promising for applications as energy storage and conversion electrodes.

As the electrochemical redox occurs simultaneously during the electrochromic and energy storage processes, it is fascinating to integrate both electrochromic and energy storage functions into a single electrode to develop an energy storage indicator, which can indicate the level of energy storage based on color changes. To prove this promising concept, we recorded the in situ response spectrum at 500 nm while performing the GCD measurement at 0.4 A g^−1^ (Fig. 3c). In the charging process, the transmittance of the NHP film gradually decreases with the color change. When charged to 0.5 V (*vs*. Ag^+^/Ag), the NHP film appears a brown-black color. In the reverse process, the color of the NHP film gradually fades away until the restoration of high transmittance at 0 V (*vs.* Ag^+^/Ag). These results confirm that developing a dual-functional energy storage indicator by the NHP film is feasible. Notably, even when the current density is as high as 2.4 A g^−1^, 40% of the optical modulation still can be reached, further validating the superior rate capability of the NHP film (Fig. 3d). The exceptional electrochromic and energy storage properties of NHP electrodes are attributed to the high electrochemical activity [19]. Additionally, the amorphous NHP film prepared by the electrodeposition method facilitates ion transport and increases accessible active sites due to the loose internal structure [47].

### Mechanism Analysis of NHP Film in Electrochromism

**Fig. 4:**
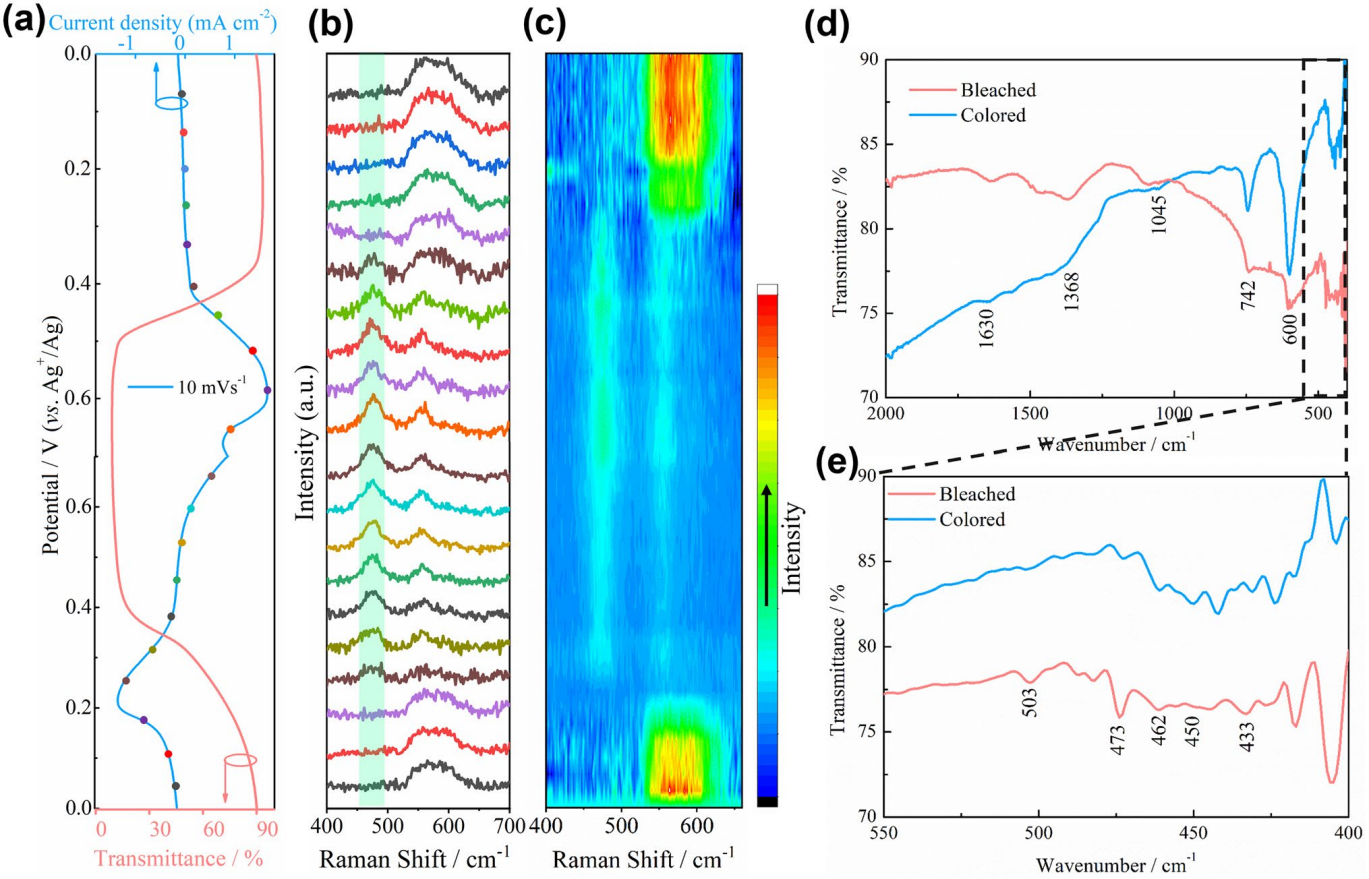
**a** CV curve at 10 mV s^−1^ in 1 M KOH electrolyte and in situ transmittance spectrum at 500 nm of the NHP film. **b** In situ Raman signal evolution and **c** chromaticity diagram of NHP film corresponding to the CV curve at 10 mV s^−1^. **d, e** Ex situ FTIR spectra of the colored and bleached NHP film

In order to understand the electrochromic mechanism of the NHP material, in situ Raman and optical transmittance spectra were conducted by applying real-time CV measurement at 10 mV s^−1^ (Fig. 4a, b). There is almost no electrochemical reaction until the potential is close to 0.4 V (*vs.* Ag^+^/Ag). At the same time, the in situ response spectrum always maintains a high transmittance, while the Raman peak of the electrode material remains unchanged, indicating that NHP film can maintain the structural integrity in KOH electrolyte. As the applied potential is higher than 0.4 V (*vs.* Ag^+^/Ag), the Raman peak of Ni^II^–O stretching vibration gradually disappears at 570 cm^−1^. Two new peaks observed at 475 and 557 cm^−1^ correspond to the Ni^III^=O and Ni^III^-O stretching vibrations, respectively, thus proving the conversion process from Ni^2+^ to Ni^3+^ [48–51]. Furthermore, the transmittance of the NHP film decreases synchronously due to the formation of Ni^3+^ species. Additionally, the intensity of the two peaks gradually increases with the continuing oxidation reaction from 0.4 to 0.7 V (*vs.* Ag^+^/Ag). In contrast, the Raman peaks at 475 and 557 cm^−1^ gradually weaken until they disappear when the applied potential decreases from 0.7 to 0.2 V (*vs.* Ag^+^/Ag). Ultimately, the broad peak at 576 cm^−1^ appears once again and the transmittance returns to the initial state from 0.2 to 0 V (*vs.* Ag^+^/Ag), demonstrating the excellent reversibility of this electrochromic NHP film. The strength and position of the Raman peak were summarized in the chromaticity diagram, which intuitively reflects component transformation and reversibility of the electrochromic process. (Fig. 4c). We further performed an ex situ FTIR analysis of the NHP film at colored and bleached states (Fig. 4d, e). Both the colored and bleached NHP films exhibit the characteristic peaks at 1630 cm^−1^ (stretching vibration of O–H in hydroxyl group), 1368 (stretching vibration of P=O), 1045 (asymmetric stretching vibration of P–O–Ni), 742 (bending vibration of P–O–H group), 503 and 473 (doubly degenerate bending mode of PO_4_^3−^) cm^−1^, indicating that HPO_4_^2−^ does not participate in the electrochromic reaction [22, 52–54]. Besides, the peaks at 450 and 462 cm^−1^ are related to the Ni–O stretching vibration, while the peak at 436 cm^−1^ is the transverse optical vibrational mode of Ni–O [50]. In particular, the stretching vibration mode belonging to higher valence (Ni^3+^–O) at 600 cm^−1^ is relatively enhanced for the colored NHP film [17, 55]. This result verifies the transition from Ni^2+^ to Ni^3+^ during the coloring process again, which is consistent with the in situ Raman spectra.

### Construction and Evaluation of NHP-Based Smart Windows

**Fig. 5:**
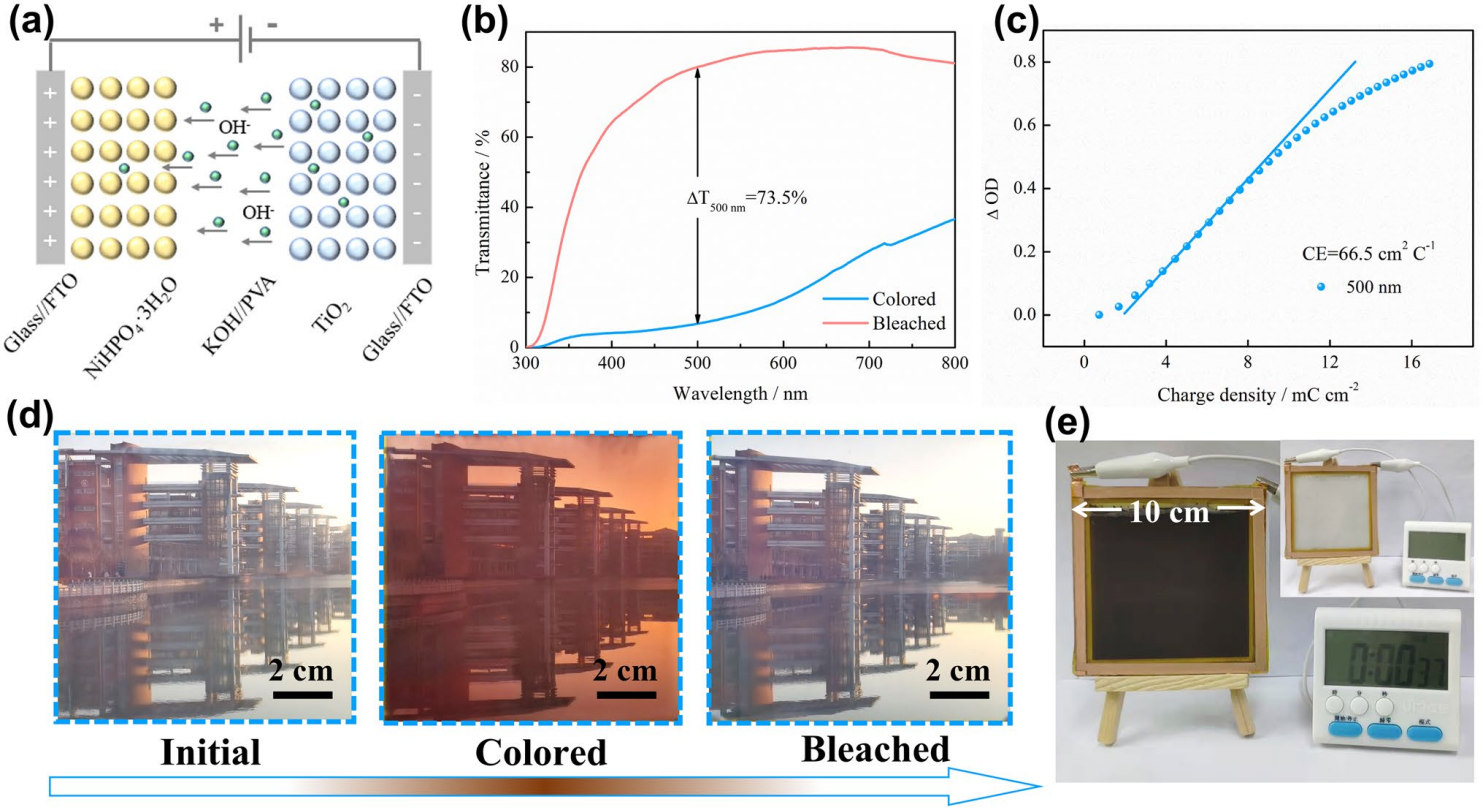
Electrochromic and energy storage performance of electrochromic energy storage device (EESD). **a** Configuration of the semi-solid device assembled by NHP film as the electrochromic layer, TiO_2_ film as the ion storage layer, and KOH/PVA gel as the semi-solid electrolyte. **b** Optical transmittance spectra of the colored (2.2 V) and bleached (− 1.5 V) EESD in the wavelength range of 300–800 nm. **c** Coloration efficiency of the as-assembled EESD. Photographs of a 100 cm^2^ EC smart window in **d** the initial, **e** colored and **f** bleached states. **g** The demo of a digital watch driven by the full charged EC smart window (Inset is the uncharged EC smart window)

We further constructed a semi-solid electrochromic energy storage device (EESD) by assembling the NHP film and TiO_2_ film with 1 M KOH/PVA gel-type electrolyte (Fig. 5a). The TiO_2_ film is an ideal counter electrode with superior charge balance ability in the KOH/PVA electrolyte and can maintain a high transmittance even if different voltages are applied (Fig. S14a, b). The CV curve and in situ optical response at 500 nm of the assembled EESD were carried out under a voltage window from − 1 to 2.2 V at 10 mV s^−1^, revealing the high reversibility of this electrochromic reaction (Fig. S15a). Additionally, the EESD delivers a large optical modulation of 73.5% at 500 nm (Fig. 5b), which is superior to reported electrochromic energy storage devices [12, 16, 40]. Moreover, the neutral coloring, fast switching time (21.6/26.6 s, Fig. S15b) and high CE (66.5 cm^2^ C^−1^, Fig. 5c) are simultaneously achieved in this EESD. It is well known that large-area EESDs are highly desirable in practical applications, especially for EC smart windows. Nevertheless, there are still tremendous challenges to be tackled in device construction. Considering the excellent electrochemical and electrochromic properties of the NHP material, we constructed a large-area EESD with a scale of 100 cm^2^. A high transmittance is observed for the device in the initial state, enabling an excellent visualization effect when served as the EC smart window (Fig. 5d). The visual field of the large-area EESD under the colored state is significantly weakened, which realizes the dynamic modulation of the incident natural light and privacy protection. The bleached state with high transmittance in Fig. 5d indicates that the EESD achieves superior reversibility, laying the foundation for its application in energy-efficient buildings. To demonstrate the energy storage properties, a single EESD with 100 cm^2^ supplies sufficient energy to drive the digital watch for nearly 40 s, illustrating its excellent charge storage performance (Fig. 5e). Moreover, the charge storage level can be predicted in real time based on the color change of the EESD, which provides many conveniences compared with traditional power sources. Thereby, the energy for driving the EC smart window can be recycled and reutilized, which is an effective approach to implementing the energy conservation. What's more, sustainable energy conversion and storage in energy-efficient buildings can be achieved by further integrating EC smart windows with solar cells, triboelectric nanogenerators, and other self-powered systems.

## Conclusions

In summary, we have developed a novel type of transition metal phosphate (NiHPO_4_·3H_2_O) for high-performance electrochromic energy storage applications. Benefiting from the high electrochemical activity, the NiHPO_4_·3H_2_O film exhibits superior electrochromic and energy storage performances, such as large optical modulation (90.8% on rigid FTO, 76.7% on flexible ITO/PET at 500 nm), high coloration efficiency (75.4 cm^2^ C^−1^ at 500 nm), rapid switching time (< 10 s) and a high specific capacity of 47.8 mAh g^−1^ at 0.4 A g^−1^. Meanwhile, the transformation mechanism of NHP film during the electrochemical process has been investigated by in situ and ex situ techniques. In addition, a large-area EC smart window of 100 cm^2^ was constructed based on the NiHPO_4_·3H_2_O electrode, which satisfied the function of effectively regulating solar radiation and protecting privacy while serving as a power source for electronic devices. We believe that the study on transition metal phosphates nanomaterials would be further extended for the applications in EC smart windows, intelligent power sources and optoelectronic devices.

### Supplementary Information

Below is the link to the electronic supplementary material.

Supplementary file1 (PDF 1336 kb)

## References

[1] Strand MT, Hernandez TS, Barile CJ, McGehee MD, Danner MG (2021). Polymer inhibitors enable >900 cm^2^ dynamic windows based on reversible metal electrodeposition with high solar modulation. Nat. Energy.

[2] Shao Z, Huang A, Ming C, Bell J, Yu P (2022). All-solid-state proton-based tandem structures for fast-switching electrochromic devices. Nat. Electron..

[3] Zhang S, Cao S, Zhang T, Fisher A, Lee JY (2018). Al^3+^ intercalation/de-intercalation-enabled dual-band electrochromic smart windows with a high optical modulation, quick response and long cycle life. Energy Environ. Sci..

[4] Zhang S, Cao S, Zhang T, Lee JY (2020). Plasmonic oxygen-deficient TiO_2-x_ nanocrystals for dual-band electrochromic smart windows with efficient energy recycling. Adv. Mater..

[5] Cai G, Darmawan P, Cheng X, Lee PS (2017). Inkjet printed large area multifunctional smart windows. Adv. Energy Mater..

[6] Yao Y, Zhao Q, Wei W, Chen Z, Zhu Y (2020). WO_3_ quantum-dots electrochromism. Nano Energy.

[7] Wen R, Granqvist CG, Niklasson GA (2015). Anodic electrochromism for energy-efficient windows: cation/anion-based surface processes and effects of crystal facets in nickel oxide thin films. Adv. Funct. Mater..

[8] Wu W, Fang H, Ma H, Wu L, Zhang W (2020). Boosting transport kinetics of ions and electrons simultaneously by Ti_3_C_2_T_x_ (MXene) addition for enhanced electrochromic performance. Nano-Micro Lett..

[9] Liang Y, Cao S, Wei Q, Zeng R, Zhao J (2021). Reversible Zn^2+^ insertion in tungsten ion-activated titanium dioxide nanocrystals for electrochromic windows. Nano-Micro Lett..

[10] Liang H, Li R, Li C, Hou C, Li Y (2019). Regulation of carbon content in MOF-derived hierarchical-porous NiO@C films for high-performance electrochromism. Mater. Horiz..

[11] Zhang L, Shi D, Liu T, Jaroniec M, Yu J (2019). Nickel-based materials for supercapacitors. Mater. Today.

[12] Lei P, Wang J, Zhang P, Liu S, Zhang S (2021). Growth of a porous NiCoO_2_ nanowire network for transparent-to-brownish grey electrochromic smart windows with wide-band optical modulation. J. Mater. Chem. C.

[13] Zeng Z, Peng X, Zheng J, Xu C (2021). Heteroatom-doped nickel oxide hybrids derived from metal-organic frameworks based on novel Schiff base ligands toward high-performance electrochromism. ACS Appl. Mater. Interfaces.

[14] Cai G, Cui P, Shi W, Morris S, Lou SN (2020). One-dimensional π-d conjugated coordination polymer for electrochromic energy storage device with exceptionally high performance. Adv. Sci..

[15] Su C, Qiu M, An Y, Sun S, Zhao C (2020). Controllable fabrication of α-Ni(OH)_2_ thin films with preheating treatment for long-term stable electrochromic and energy storage applications. J. Mater. Chem. C.

[16] Xue J, Wang S, Zhang H, Song Y, Li Y (2020). N-doped two-dimensional ultrathin NiO nanosheets for electrochromic supercapacitor. J. Mater. Sci. Mater. Electron..

[17] Cai G, Wang X, Cui M, Darmawan P, Wang J (2015). Electrochromo-supercapacitor based on direct growth of NiO nanoparticles. Nano Energy.

[18] Wang J, Li F, Zhu F, Schmidt OG (2018). Recent progress in micro-supercapacitor design, integration, and functionalization. Small Methods.

[19] Huang J, Xiong Y, Peng Z, Chen L, Wang L (2020). A general electrodeposition strategy for fabricating ultrathin nickel cobalt phosphate nanosheets with ultrahigh capacity and rate performance. ACS Nano.

[20] Zhao X, Kong X, Liu Z, Li Z, Xie Z (2021). The cutting-edge phosphorus-rich metal phosphides for energy storage and conversion. Nano Today.

[21] Septiani NLW, Kaneti YV, Fathoni KB, Wang J, Ide Y (2020). Self-assembly of nickel phosphate-based nanotubes into two-dimensional crumpled sheet-like architectures for high-performance asymmetric supercapacitors. Nano Energy.

[22] Sun Z, Yuan M, Lin L, Yang H, Li H (2019). Needle grass-like cobalt hydrogen phosphate on Ni foam as an effective and stable electrocatalyst for the oxygen evolution reaction. Chem. Commun..

[23] Jing C, Song X, Li K, Zhang Y, Liu X (2020). Optimizing the rate capability of nickel cobalt phosphide nanowires on graphene oxide by the outer/inter-component synergistic effects. J. Mater. Chem. A.

[24] Wang Z, Wu Y, Cui M, Ji S, Wang H (2021). 1D NiHPO_4_ nanotubes prepared using dissolution equilibrium as bifunctional electrocatalyst for high-efficiency water splitting. J. Power Sources.

[25] Wang Z, Chen F, Kannan P, Ji S, Wang H (2019). Nickel phosphate nanowires directly grown on Ni foam as binder-free electrode for pseudocapacitors. Mater. Lett..

[26] Lo IH, Wang JY, Huang KY, Huang JH, Kang WP (2016). Synthesis of Ni(OH)_2_ nanoflakes on ZnO nanowires by pulse electrodeposition for high-performance supercapacitors. J. Power Sources.

[27] Tian Y, Li Z, Dou S, Zhang X, Zhang J (2018). Facile preparation of aligned NiO nanotube arrays for electrochromic application. Surf. Coat. Technol..

[28] Zhang Y, Cui Q, Zhang X, McKee WC, Xu Y (2016). Amorphous Li_2_O_2_: chemical synthesis and electrochemical properties. Angew. Chem. Int. Ed..

[29] Han X, Li J, Lu J, Luo S, Wan J (2021). High mass-loading NiCo-LDH nanosheet arrays grown on carbon cloth by electrodeposition for excellent electrochemical energy storage. Nano Energy.

[30] Fa D, Yu B, Miao Y (2019). Synthesis of ultra-long nanowires of nickel phosphate by a template-free hydrothermal method for electrocatalytic oxidation of glucose. Colloids Surf. A.

[31] Sun T, Shen L, Jiang Y, Ma J, Lv F (2020). Wearable textile supercapacitors for self-powered enzyme-free smartsensors. ACS Appl. Mater. Interfaces.

[32] Yang P, Sun P, Mai W (2016). Electrochromic energy storage devices. Mater. Today.

[33] Zhang G, Hu J, Nie Y, Zhao Y, Wang L (2021). Integrating flexible ultralight 3D Ni micromesh current collector with NiCo bimetallic hydroxide for smart hybrid supercapacitors. Adv. Funct. Mater..

[34] Zhao Q, Wang J, Ai X, Pan Z, Xu F (2021). Large-area multifunctional electro-chromic-chemical device made of W_17_O_47_ nanowires by Zn^2+^ ion intercalation. Nano Energy.

[35] Wang Y, Wang S, Wang X, Zhang W, Zheng W (2019). A multicolour bistable electronic shelf label based on intramolecular proton-coupled electron transfer. Nat. Mater..

[36] Cao F, Pan GX, Xia XH, Tang PS, Chen HF (2013). Hydrothermal-synthesized mesoporous nickel oxide nanowall arrays with enhanced electrochromic application. Electrochim. Acta.

[37] Yuan Y, Xia X, Wu J, Chen Y, Yang J (2011). Enhanced electrochromic properties of ordered porous nickel oxide thin film prepared by self-assembled colloidal crystal template-assisted electrodeposition. Electrochim. Acta.

[38] Zhu L, Ong WL, Lu X, Zeng K, Fan HJ (2017). Substrate-friendly growth of large-sized Ni(OH)_2_ nanosheets for flexible electrochromic films. Small.

[39] Hou S, Gavrilyuk AI, Zhao J, Geng H, Li N (2018). Controllable crystallinity of nickel oxide film with enhanced electrochromic properties. Appl. Surf. Sci..

[40] Zhao Y, Zhang X, Chen X, Li W, Wang L (2021). Preparation of Sn-NiO films and all-solid-state devices with enhanced electrochromic properties by magnetron sputtering method. Electrochim. Acta.

[41] Chen Y, Wang Y, Sun P, Yang P, Du L (2015). Nickel oxide nanoflake-based bifunctional glass electrodes with superior cyclic stability for energy storage and electrochromic applications. J. Mater. Chem. A.

[42] Ren Y, Zhou X, Zhang H, Lei L, Zhao G (2018). Preparation of a porous NiO array-patterned film and its enhanced electrochromic performance. J. Mater. Chem. C.

[43] Zhou S, Wang S, Zhou S, Xu H, Zhao J (2020). An electrochromic supercapacitor based on an MOF derived hierarchical-porous NiO film. Nanoscale.

[44] Luo Z, Liu L, Yang X, Luo X, Bi P (2020). Revealing the charge storage mechanism of nickel oxide electrochromic supercapacitors. ACS Appl. Mater. Interfaces.

[45] Zhao C, Du F, Wang J (2015). Flower-like nickel oxide micro/nanostructures: synthesis and enhanced electrochromic properties. RSC Adv..

[46] Cai G, Chen J, Xiong J, Lee-Sie A, Wang J (2020). Molecular level assembly for high-performance flexible electrochromic energy-storage devices. ACS Energy Lett..

[47] Huo X, Zhang H, Shen W, Miao X, Zhang M (2019). Bifunctional aligned hexagonal/amorphous tungsten oxide core/shell nanorod arrays with enhanced electrochromic and pseudocapacitive performance. J. Mater. Chem. A.

[48] Wang R, Liu H, Zhang K, Zhang G, Lan H (2021). Ni(II)/Ni(III) redox couple endows Ni foam-supported Ni_2_P with excellent capability for direct ammonia oxidation. Chem. Eng. J..

[49] Hu C, Hu Y, Fan C, Yang L, Zhang Y (2021). Surface-enhanced Raman spectroscopic evidence of key intermediate species and role of NiFe dual-catalytic center in water oxidation. Angew. Chem. Int. Ed..

[50] Zhang J, Cai G, Zhou D, Tang H, Wang X (2014). Co-doped NiO nanoflake array films with enhanced electrochromic properties. J. Mater. Chem. C.

[51] Gao P, Zeng Y, Tang P, Wang Z, Yang J (2021). Understanding the synergistic effects and structural evolution of Co(OH)_2_ and Co_3_O_4_ toward boosting electrochemical charge storage. Adv. Funct. Mater..

[52] Sronsri C, Danvirutai C, Noisong P (2017). Double function method for the confirmation of the reaction mechanism of LiCoPO_4_ nanoparticle formation, reliable activation energy, and related thermodynamic functions. React. Kinet. Mech. Cat..

[53] Qiu W, Xiao H, Yu M, Li Y, Lu X (2018). Surface modulation of NiCo_2_O_4_ nanowire arrays with significantly enhanced reactivity for ultrahigh-energy supercapacitors. Chem. Eng. J..

[54] Zhang Y, Shi J, Cheng C, Zong S, Geng J (2018). Hydrothermal growth of Co_3_(OH)_2_(HPO_4_)_2_ nano-needles on LaTiO_2_N for enhanced water oxidation under visible-light irradiation. Appl. Catal. B.

[55] Ren Y, Chim WK, Guo L, Tanoto H, Pan J (2013). The coloration and degradation mechanisms of electrochromic nickel oxide. Sol. Energy Mater. Sol. Cells.

