# Supplementary material for: An Electrochromic Nickel Phosphate Film for Large-Area Smart Window with Ultra-Large Optical Modulation
Source: Nanomicro Lett. 2023 Jan 11;15:34. doi: 10.1007/s40820-022-01002-4 (PMC9834494; doi:10.1007/s40820-022-01002-4)
Supplement: Supplementary file 1 — Supplementary file1 (PDF 1336 kb) [file 40820_2022_1002_MOESM1_ESM.pdf]

Supporting Information for

An Electrochromic Nickel Phosphate Film for Large-Area Smart Window with Ultra-Large Optical Modulation

Pengyang Lei[1, 2, †], Jinhui Wang[1, †], Yi Gao[1], Chengyu Hu[1], Siyu Zhang[1], Xingrui Tong[1], Zhuanpei Wang[1], Yuanhao Gao[2, *], Guofa Cai[1, *]

[1]Key Laboratory for Special Functional Materials of Ministry of Education, National & Local Joint Engineering Research Center for High-efficiency Display and Lighting Technology, School of Materials and Engineering, and Collaborative Innovation Center of Nano Functional Materials and Applications, Henan University, Kaifeng 475004, P. R. China

[2]Key Laboratory of Micro-Nano Materials for Energy Storage and Conversion of Henan Province, Institute of Surface Micro and Nano Materials, College of Chemical and Materials Engineering, Xuchang University, Xuchang, Henan, 461000, P. R. China

[†]Pengyang Lei and Jinhui Wang contributed equally to this work.

*Corresponding authors. E-mail: gyh-2007@sohu.com (Yuanhao Gao); caiguofa@henu.edu.cn (Guofa Cai)

Supplementary Figures

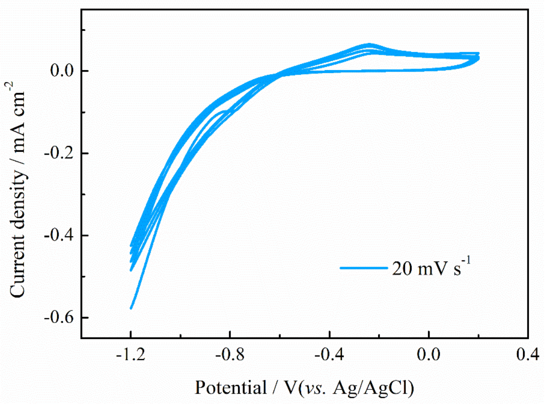

Fig. S1 The deposition curve of NHP nanoparticles on FTO substrate by a typical electrochemical deposition method (between -1.2 and 0.2 V *vs.* Ag/AgCl, 20 mV s^{-1})

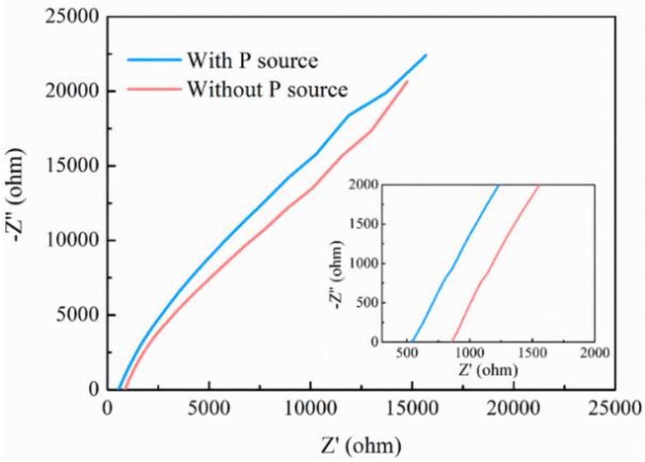

Fig. S2 Nyquist plots of electrolytes for electrodeposition with or without phosphorus source (inset: partial enlargement). The ionic conductivity (σ) was calculated from the electrolyte resistance (*R*), according to Eq. [S1]:

$$\sigma = \frac{1}{R} * \frac{l}{A} \qquad \text{(S1)}$$

Here, *A* refer to cross-sectional area of the electrolyte.

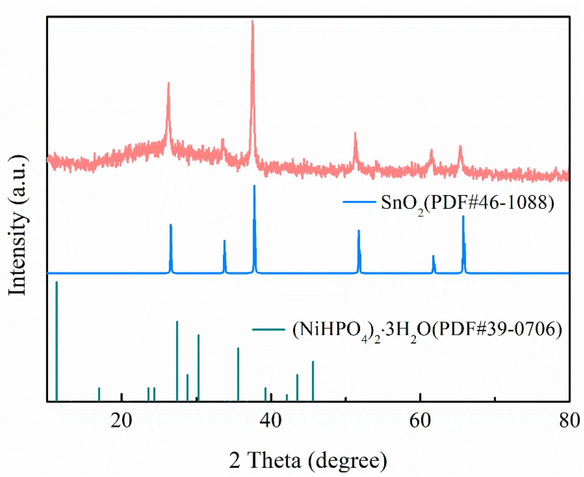

Fig. S3 XRD pattern of NHP nanoparticles film on FTO substrate

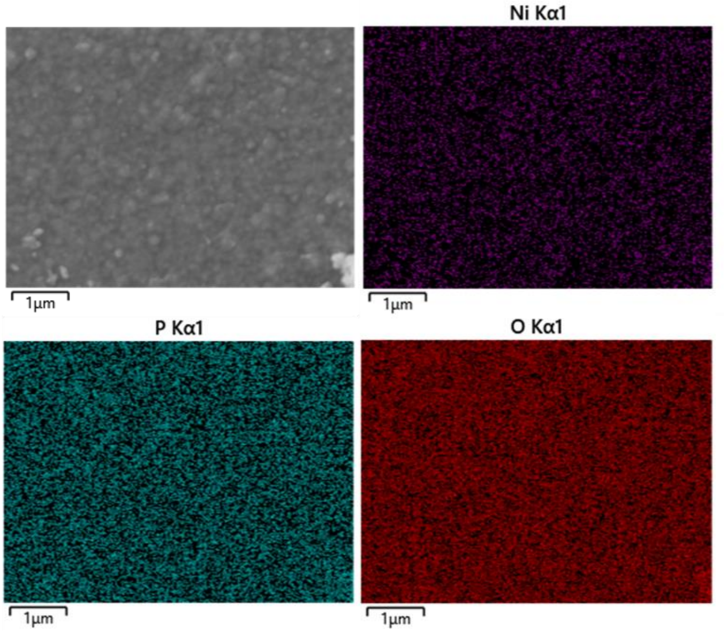

Fig. S4 Elemental mapping of Ni, P and O was obtained by SEM for NHP nanoparticles

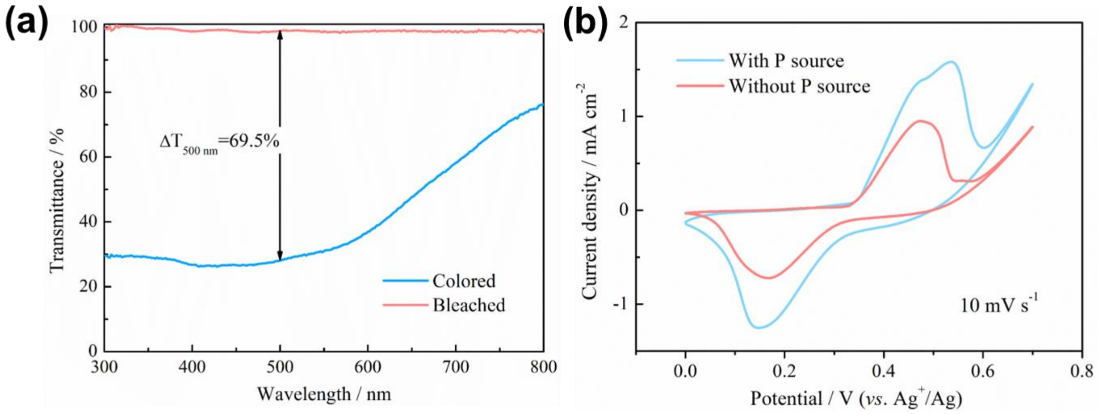

Fig. S5 a Optical transmittance spectra of the phosphorus-free film in 300-800 nm at colored (0.7 V *vs.* Ag^+/Ag) and bleached (0 V *vs.* Ag^+/Ag) states. **b** Comparison of the electrochemical activity of electrode materials deposited 4 cycles from -1.2 to 0.2 V (*vs.* Ag/AgCl) at 20 mV s^{-1} in the different electrolytes (with or without phosphorus source)

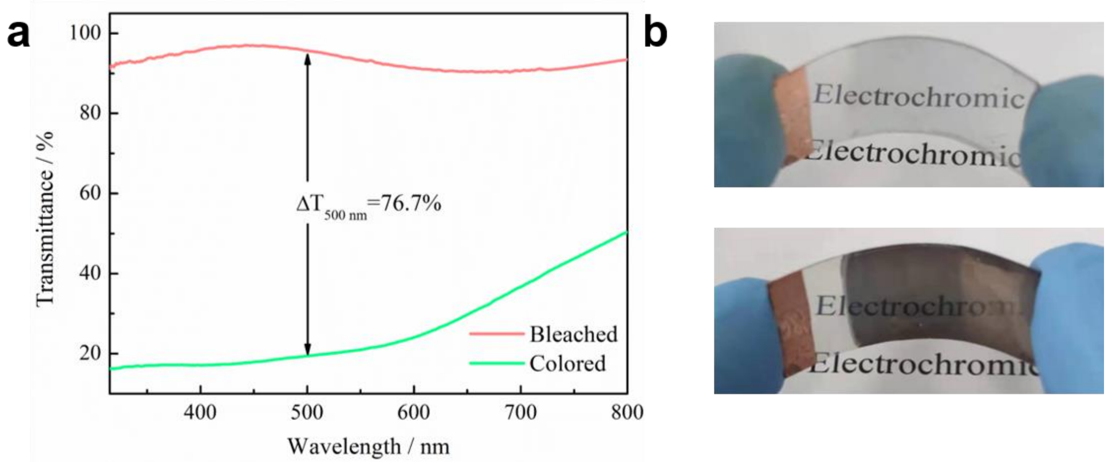

Fig. S6 a The optical transmittance spectra of NHP film on flexible ITO/PET substrate in 300-800 nm at colored (0.8 V *vs.* Ag^{+}/Ag) and bleached (-0.1 V *vs.* Ag^{+}/Ag) states. **b** Digital photographs of NHP film in the colored and bleached state under bending

Table S1 Comparison of optical modulation at 500 nm and switching time of NHP films at different electrodeposition cycles

Sample	$\Delta T_{500\ nm}$(%)	t_c(s)	t_b(s)
2cycles	73.5	5.4	7.8
4cycles	90.8	7.1	9.6
6cycles	88.3	8.3	12.9
8cycles	86.9	8.7	16.9

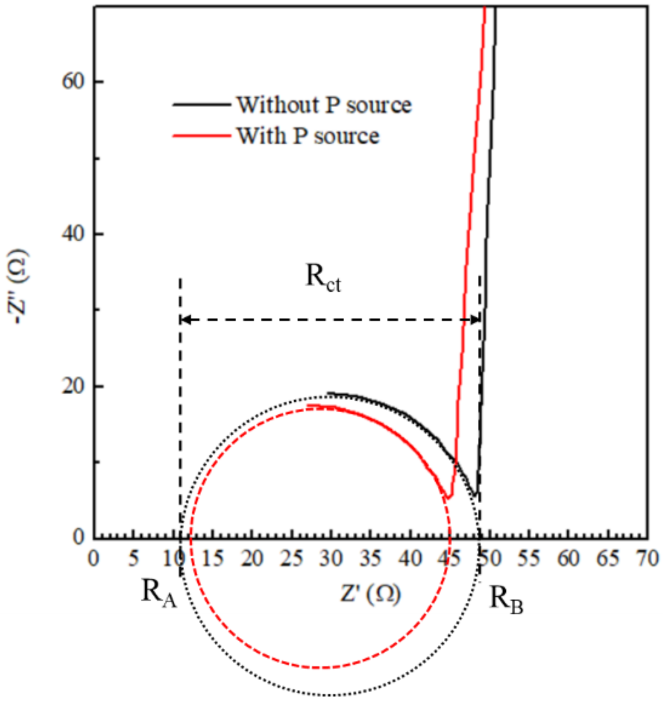

Fig. S7 Nyquist plots of materials prepared by electrodeposition with or without phosphorus source. (R_{ct} = R_B -R_A, the R_A, R_B and R_{ct} represent electrolyte resistance, internal resistance and charge transfer resistance, respectively)

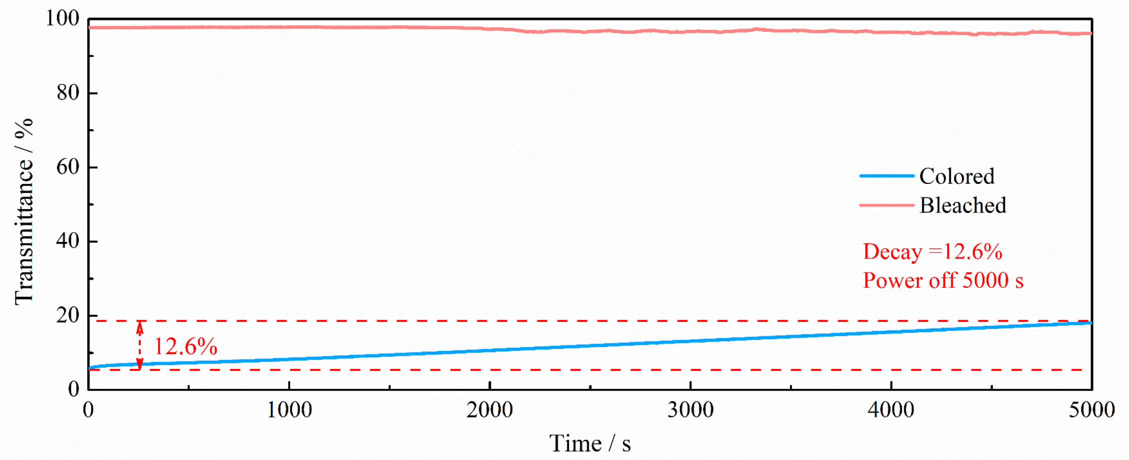

Fig. S8 Optical memory at 500 nm of the NHP film at stimulation voltages of 0 and 0.7 V (*vs.* Ag^+/Ag) for 100 s, power off for 5000 s

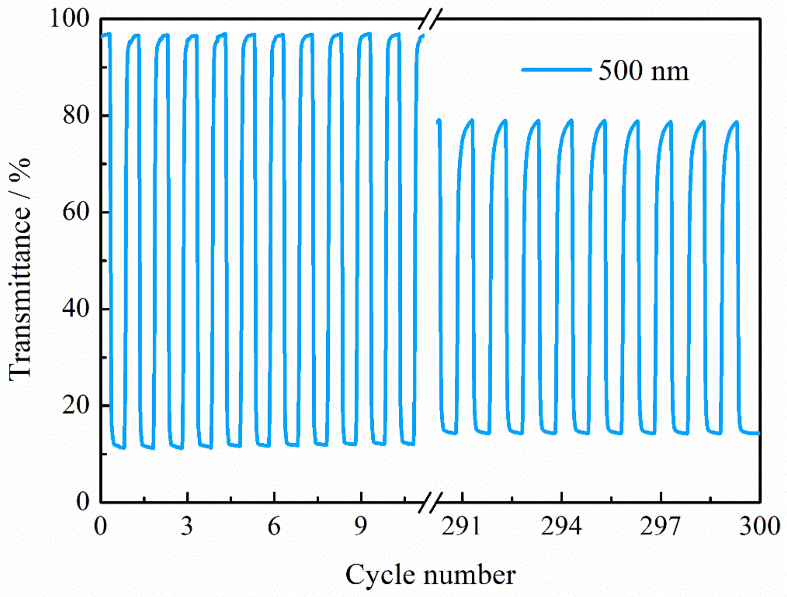

Fig. S9 Cyclic stability measurement of NHP film at 500 nm by chronoamperometry and in-situ spectroscopic response

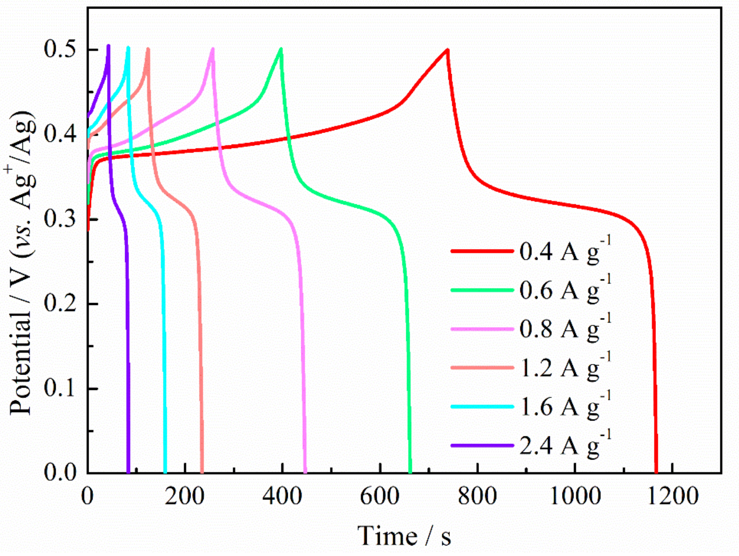

Fig. S10 The galvanostatic charge-discharge profiles of NHP film from 0 to 0.5 V (*vs.* Ag^+/Ag) under different current densities

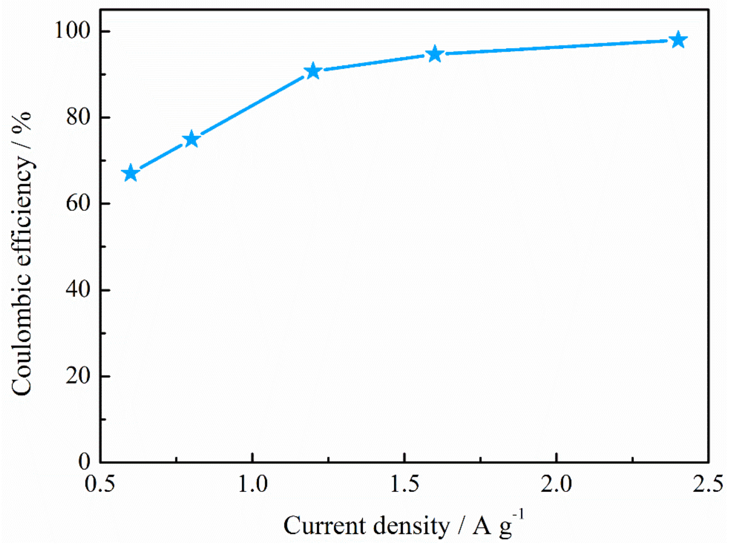

Fig. S11 Coulombic efficiency of the NHP film corresponding to different current densities

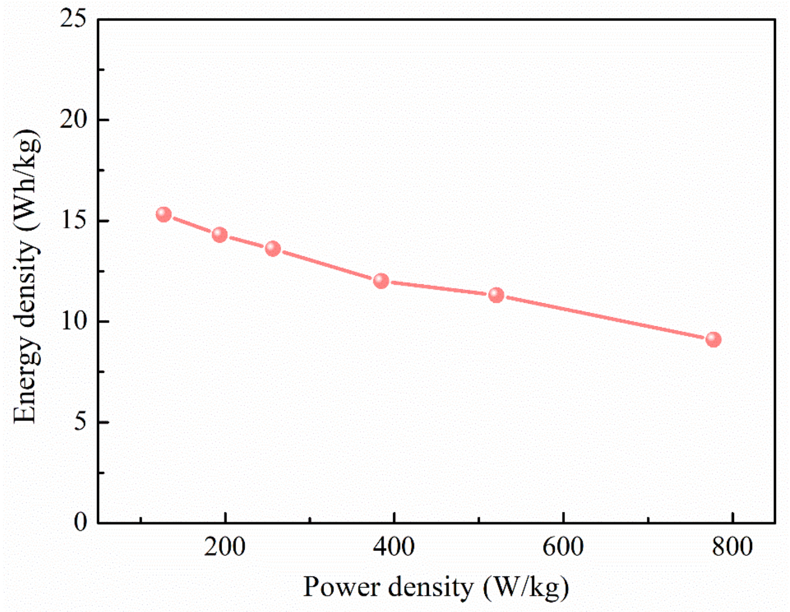

Fig. S12 Ragone plot of the NHP film at different current densities

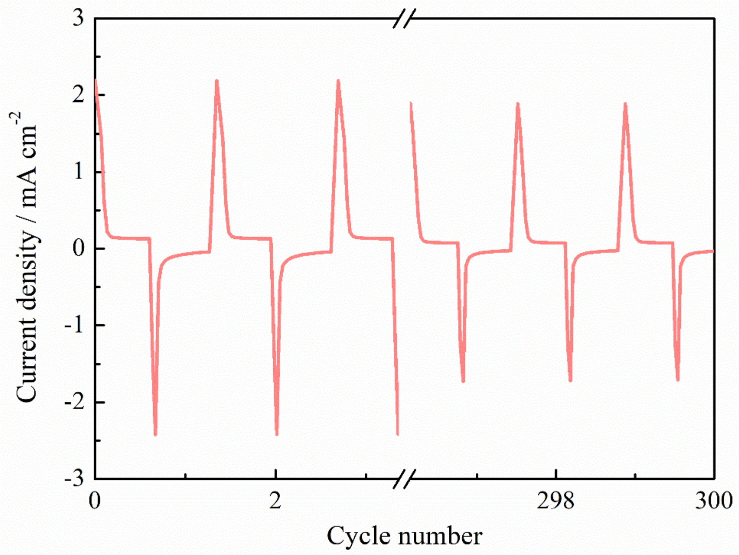

Fig. S13 The potentiostatic charge-discharge profiles of NHP film by alternately applying 0 and 0.7 V (*vs.* Ag^+/Ag) each for 30 s

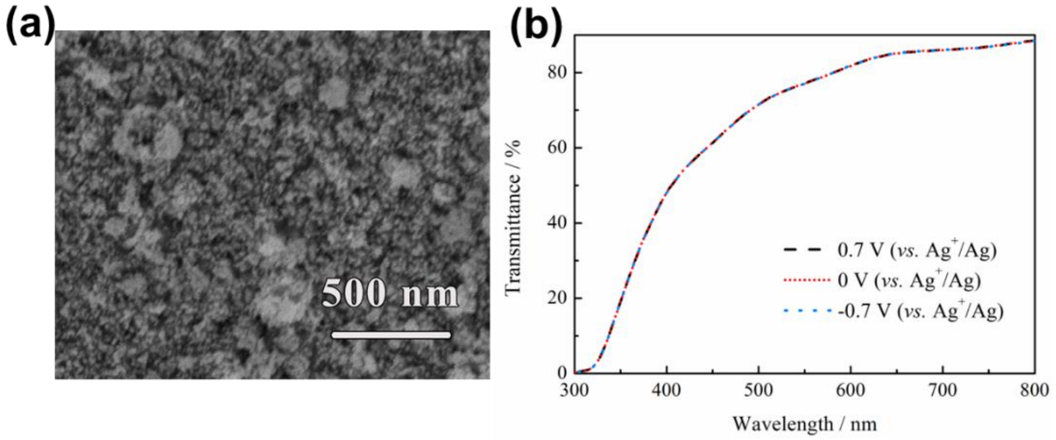

Fig. S14 a SEM image of the electrostatic sprayed TiO_2 nanoparticles film. **b** The transmittance spectra of TiO_2 nanoparticles film in 1 M KOH/PVA electrolyte under different potentials

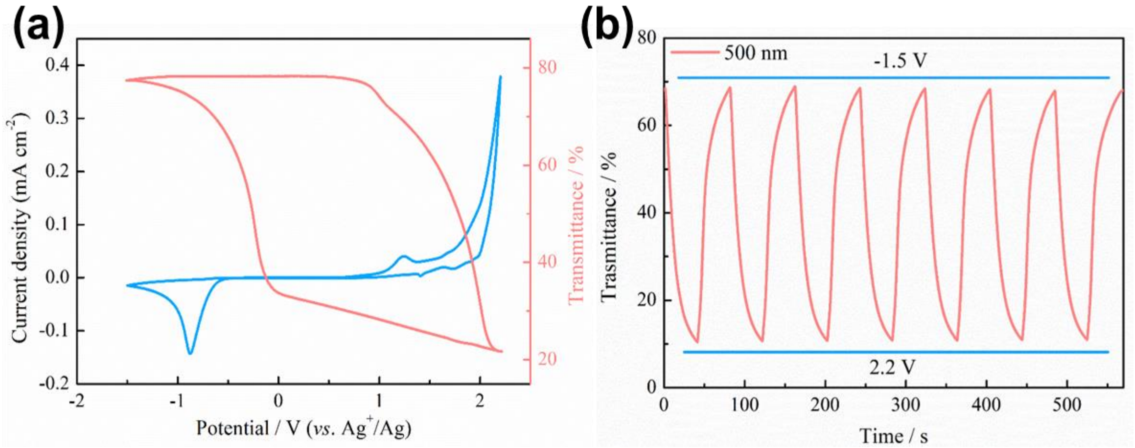

Fig. S15 a CV property and in-situ optical response at 500 nm of the assembled device was tested between -1.5 and 2.2 V at 10 mV s^{-1}. **b** The in-situ transmittance response at 500 nm was obtained by applying square wave voltages of 2.2 V and -1.5 V for 40 s

Supplementary Reference

[S1]Z. Wang, B. François, Implementation of a choline bis(trifluoromethylsulfonyl)imide aqueous electrolyte for low temperature EDLCs enabled by a cosolvent. J. Energy Chem. **70**, 84-94 (2022). https://doi.org/10.1016/j.jechem.2022.01.022
